# Effect of *Cymbopogon* olivieri-based herbal vaginal product on bacterial vaginosis

**DOI:** 10.1590/1806-9282.20231788

**Published:** 2024-07-19

**Authors:** Mitra Esmaili, Zarrin Sarhadynejad, Zohreh Salari, Tania Dehesh, Mahdiyeh Lashkarizadeh, Haleh Tajadini, Mohadese Kamali

**Affiliations:** 1 Kerman University of Medical Sciences, Faculty of Persian Medicine, Herbal and Traditional Medicines Research Center, Department of Traditional Medicine – Kerman, Iran.; 2 Kerman University of Medical Sciences, Faculty of Persian Medicine, Herbal and Traditional Medicines Research Center, Department of Traditional Pharmacy – Kerman, Iran.; 3 Kerman University of Medical Sciences, Afzalipour School of Medicine, Obstetrics and Gynecology Center – Kerman, Iran.; 4 Kerman University of Medical Sciences, School of Public Health, Department of Biostatistics and Epidemiology – Kerman, Iran.; 5 Kerman University of Medical Sciences, School of Medicine, Department of Pathology and Stem Cell Research Center – Kerman, Iran.; 6 Kerman University of Medical Sciences, Faculty of Persian Medicine, Medical Mycology and Bacteriology Research Center, Department of Traditional Medicine – Kerman, Iran.

**Keywords:** Bacterial vaginitis, Metronidazole, Medicine, Persian, Herbal medicine, Lemon grass

## Abstract

**OBJECTIVE:**

Bacterial vaginosis is the most common vaginal infection in reproductive-age women. If it is not treated, the quality of life will be reduced. In this study, the herbal medicine product *Cymbopogon olivieri* was used for its treatment.

**METHODS:**

This study was conducted with 90 women. The patients were randomly divided into two groups of 45: *Cymbopogon olivieri* and metronidazole. The treatment period was 7 days for each group. Improvement status was determined by eliminating at least three out of four of Amsel's criteria. A new variable with two order levels (negative and positive) was constructed. This new variable shows the status of the treatment process. Chi-square and Fisher's exact tests were used to examine the relationship between the new variable and treatment status.

**RESULTS:**

The results demonstrate that *Cymbopogon olivieri* and metronidazole significantly reduced the burning, itching, malodor, abnormal vaginal discharge, pH, clue cell, and positive whiff test (p<0.05). The findings also demonstrate that neither treatment was statistically different from the other for at least three of Amsel's criteria.

**CONCLUSION:**

*This study shows that the effect of Cymbopogon olivieri on bacterial vaginosis is similar to that of metronidazole. Hence, Cymbopogon olivieri* is a suitable option to treat bacterial vaginosis.

## INTRODUCTION

Bacterial vaginosis (BV) is characterized by the loss of normal vaginal flora and the overgrowth of facultative anaerobic bacteria^1^. BV is the most common form of vaginal infection in women of reproductive age worldwide^2^, and it has been reported from 20 to 60% in different populations^3^. The most common symptom of BV is a bad odor from vaginal discharge^4^. Vaginal examination shows a gray, thin, and homogeneous discharge covering the vaginal walls^5^. Risk factors include low socioeconomic status, poor hygiene, early sexual activity, multiple sexual partners, psychological stress, and biogenetic factors^3^. Metronidazole (orally or vaginally) is the first line of therapy^6^. However, its side effects (nausea, vomiting, abdominal pain, or diarrhea)^7-9^ and traditional use of herbal medicines have encouraged researchers to investigate the effectiveness and safety of herbal medicines. Due to its better cultural acceptability, greater adaptability to the human body, and fewer side effects, herbal therapy is the main treatment method for primary care for about 75% of the world's population, especially in developing and developed countries^10^. However, in evidence-based medicine, the traditional use of herbs and expert opinion are considered the lowest level of evidence for the safety and efficacy of drugs. The highest level of evidence comes from randomized clinical trials and unbiased systematic reviews, with or without meta-analysis^11^. One of the plants whose antimicrobial and antioxidant effects have been shown in non-clinical studies is Lemongrass [*Cymbopogon olivieri (Boiss.*)]^12^. *Cymbopogon* genus is a member of the family of *Gramineae* ^13^. Mahboubi and Kazempour^13^ and Tibenda et al.^14^ showed these effects for this plant. As no clinical study has investigated the effect of this plant on BV, *C. olivieri* was selected for this clinical trial.

## METHODS

### Study design

This study was a clinical trial using the double-blind, randomized, quadraplexes one-to-one block method, which was designed by the epidemiologist and conducted on married women aged 18–50 years in Kerman, Iran, from December 2021 to November 2022. The trial participants were divided into two groups: *C. olivieri* and metronidazole. It was performed following Consolidated Standards of Reporting Trials (CONSORT) guidelines.

### Ethical consideration

The trial protocol adhered to the Declaration of Helsinki criteria and was approved by the Medical Ethics Committee of Kerman University (No. IR.KMU.REC.1400.547). It was also registered with the Iranian Registry of Clinical Trials (No. IRCT20211111053036N1).

### Preparation of herbal medicine

*Cymbopogon olivieri* was collected from farms in Kerman (30.3°N, 57.0°E) from March to April 2021. An herbalist verified the authenticity of the plant. Herbarium number in the Natural Resources Research Center in Kerman, Iran, is 5662. A dried hydroalcoholic extract of *C. olivieri* (ethanol 70%) was utilized to make the formulation. Samples underwent standardization testing and microbiological contamination research. Similar dose forms, hard gelatin capsules containing powdered metronidazole, were utilized to blind the medications. The final preparations were labeled and coded. Researchers and patients did not know the contents of the packages.

### Assessment of microbial contamination

The microbial contamination was evaluated with the total number of live microorganisms^15^.

### Determining the amount of essential oil and plant extract

The essential oil was prepared from the dried leaves and stem of *C. olivieri* by the method of hydro distillation with a Clevenger apparatus.

### Total polyphenol content determination

This test was performed on plant extract by the Folin-Ciocalteu method, and the standard solution of gallic acid (in concentrations of 12.5, 25, 50, 100, and 200 μg/mL) was used for calibration curve^15^.

### Determination of total ash and moisture content and pH

The usual methods of The United States Pharmacopeia were used for determining of total ash and moisture content of the prepared formulation. Also, a pH meter was used to measure the acidity or alkalinity of the aqueous solution of final preparation^15^.

### GC/MS analysis

The gas chromatography device used in the study was an Agilent 6890 type with specific column specifications. The essential oil sample was diluted and injected into the GC/MS machine, and the temperature was controlled in a specific manner during the analysis. The mass spectrometer used was an Agilent 5973 model with specific settings. The spectra obtained were compared with reference books and articles to identify the components of the essential oil^13^.

### Sample size

A pilot study with 60 patients was conducted (30 patients in each group). The final sample size was calculated based on the results of the pilot study, with 90 patients (45 patients in each group). The outcome of the sample size calculation was the number of improved patients according to at least three symptoms. In fact, the patients who have an improvement in at least three symptoms (burning, itching, malodor, abnormal vaginal discharge, pH, clue cell, and positive whiff test) were considered improved. The number of improved patients is the outcome of the calculation of the final sample size. The sample size was calculated with the PASS software version 15.

### Randomization and allocation concealment

The mechanism for implementing the allocation sequence was performed with sealed envelopes. The doctor visited the patient, opened the envelope, and allocated the code to the patient. Then, the patient was referred to the nurse and received the drug according to the code. The patient's name and code were registered. The doctor and the nurse did not know the content of the codes and drugs. Drugs had exactly a similar shape in both codes. We tried to remind the patient about the importance of treatment by phone every day.

### Inclusion and exclusion criteria

Inclusion criteria were as follows: married women between 18 and 50 years old, ready to go for examinations, consent to participate in the study, and not using antibiotics and other vaginal creams or suppositories in the last 2 weeks before entering the study. Exclusion criteria were as follows: any complications during treatment, pregnancy, or breastfeeding; having a complex and recurrent BV infection diagnosed by a gynecologist; the presence of chronic diseases; use of immunosuppressants or immunodeficiency disease; and use of oral contraceptive pill (OCP).

### Intervention

The patients in the herbal product group were advised to use one hard gelatin capsule, size 00, containing 500 mg hydroalcoholic extract of *C. olivieri* vaginally with an applicator at bedtime for seven nights. The other group received 500 mg metronidazole powder capsules like the herbal product group. The demographic data of patients were entered into a pre-made checklist. After history-taking, vaginal samples were taken in a lithotomy position with a disposable, sterile specimen without lubricant. The vagina and cervix of each patient were examined by a gynecologist and any abnormal evidence was noted in the checklist. Using a sterile swab, samples were taken from the top portion of the vagina's lateral wall, and the discharge specimen was then put on two slides. The pathology lab received one slide with a fixative on it for microscopic examination. The second specimen was mixed with one drop of potassium hydroxide (KOH) at 10%. The pH meter paper with a range of 1–14 made by Merck Germany Company was used to measure the pH. The researcher tested the vaginal pH 1 min after the pH-meter strip contacted the vaginal wall. Clinical criteria were entered in the checklist too. The researcher changed the color and then compared it with the box's regular color. A gynecologist confirmed the uniformity of the vaginal samples. Every day, a phone call was used to inquire about the patients' symptom intensity, which was then noted in the checklist. The patients returned 10 days after the completion of the treatment. The clinical criteria and the patient's complaints were re-evaluated.

### Outcome measures

Clinical diagnosis of BV was based on Amsel's criteria, which include pH>4.5, a positive whiff test, grayish-white homogeneous discharge, and the presence of Clue cells >20%^16^. Abnormal evidence, any inflammation, and vaginal discharge were examined in terms of color, texture, and malodor. Outcome measures were burning, itching, malodor, abnormal vaginal discharge, pH, clue cell, and positive whiff test. The absence of at least three of Amsel's criteria after the end of treatment was considered treatment improvement, and any other result was considered treatment failure.

### Statistical analysis

The outcome variable was categorical with two levels (yes and no), the postcode was subtracted from the previous code, and a new variable with two order levels (negative and positive) was constructed. This new variable shows the status of the treatment process. Chi-square and Fisher's exact tests were used to examine the relationship between the new variable and treatment status.

## RESULTS

### Evaluation of herbal medicine microbial contamination

Microbial tests were run on the final formulation after extracting and preparing the final sample. The findings revealed that *C. olivieri*'s fungal and microbial contamination levels were within The United States Pharmacopeia's permissible limit. Also, the absence of pathogenic pathogens, especially *Candida albicans, Pseudomonas aeruginosa, and Staphylococcus aureus*, in the final product of *C. olivieri* was confirmed.

### Amount of essential oil and extractable matter

Notably, 2.5 mL of essential oil was obtained per 100 g of the dried plant (2.5% w/v) and 11 g of hydroalcoholic extract was obtained per 100 g of the dry mass of the plant (11% w/w).

### Physicochemical evaluation of the final product of Cymbopogon olivieri

pH is 5.33, total ash is 13.25%, and the moisture content in the final product of *C. olivieri* is 9.59% w/w.

### Determination of the total amount of phenolic compounds based on gallic acid in the final product of Cymbopogon olivieri

The total amount of phenols in the final product of *C. olivieri* was 88/86±6/50 mg gallic acid/g.

### Analysis of essential oil of Cymbopogon olivieri

Analysis was performed by gas chromatography and GC/MS mass spectrometer. The analysis is shown in Table 1. The most components are in order: β-Eudesmol (14.42%), ρ-2-menthen (11.84%), Elemol (9.81%), and Agarospirol (6.49%).

**Table 1:** Gas chromatography-mass spectrometry (GC/MS) analysis.

No.	RT	%	Components	KI	Type
1	10.75	0.14	Tricyclene	926	MH
2	11.29	0.25	α-Pinene	939	MH
3	12.17	0.58	Camphene	954	MH
4	14.67	2.8	δ-2-Carene	1002	MH
5	15.13	0.73	α-Phellandrene	1003	MH
6	15.66	0.29	α-Terpinene	1017	MH
7	16.16	0.95	ρ-Cymene	1024	MH
8	16.32	1.12	Limonene	1029	MH
9	16.44	1.69	β-Phellandrene	1029	MH
10	16.66	0 16	(E)-β-Ocimene	1050	MH
11	19.72	0.07	ρ-Cymenene	1091	MH
12	20.08	0.14	Linalool	1096	MO
13	21.21	0.06	Fenchol	1119	MO
14	21.44	11.84	ρ-2-Menthen-l-ol	1119	MO
15	22.07	0.1	trans-ρ-Mentha-2,8-dien-1 -ol	1122	MO
16	22.38	7.41	1-Terpineol	1133	MO
17	23.93	0.93	ρ-Mentha-1,5-dien-8-ol	1170	MO
18	24.25	0.09	Terpinen-4-ol	1177	MO
19	24.75	0.62	ρ-Cymen-8-ol	1182	MO
20	25.06	3.89	Cis-Piperitol	1198	MO
21	25.69	4.48	Trans-Piperitol	1208	MO
22	27.94	1.11	Piperitone	1252	MO
23	31.68	0.05	α-Cubebene	1351	SH
24	33.02	0.22	α-Copaene	1376	SH
25	33.38	0.16	β-Bourbonene	1388	SH
26	33.62	1.03	β-Elemene	1390	SH
27	34.97	0.08	(E)-Caryophyllene	1419	SH
28	36.38	0.05	β-Barbatene	1442	SH
29	37.32	0.38	γ-Muurolene	1479	SH
30	37.61	0.73	Germacrene D	1481	SH
31	37.73	0.67	4, 1 1-selinadiene	1485	SH
32	37.99	0.66	β-Selinene	1490	SH
33	38.08	0.66	Valencene	1496	SH
34	38.26	0.77	α-Selinene	1498	SH
35	38.72	0.12	α-Chamigrene	1503	SH
36	38.87	0.25	Cuparene	1504	SH
37	38.94	0.25	γ-Cadinene	1513	SH
38	39.08	0.92	δ-Cadinene	1523	SH
39	39.23	0.35	7-epi-α-Sclinene	1526	SH
40	39.35	0.21	Cis-Calamenene	1532	SH
41	40.43	9.81	Elemol	1549	SO
42	41.83	0.13	Caryophyllene oxide	1583	SO
43	42.79	2.17	Eudesmol <5-epi-7-epi-α->	1607	SO
44	43.75	2.84	γ-Eudesmol	1632	SO
45	43.89	6.49	Agarospirol	1642	SO
46	44.25	1.34	Hinesol	1641	SO
47	44.72	14.42	β-Eudesmol	1650	SO
48	44.92	0.97	7-epi-α-Eudesmol	1663	SO
49	45.12	9.49	β-Maaliene	1671	SH
		94.67	Total Identified		

MH: monoterpene hydrocarbons; MO: oxygenated monoterpenes; SH: sesquiterpene hydrocarbons; SO: oxygenated sesquiterpene.

### Evaluation of capsule disintegration time

The disintegration time of *C. olivieri* in water is 7 min, and in pH 4.6, it is 7 min.

### Baseline and demographic characteristics

**Figure 1:**
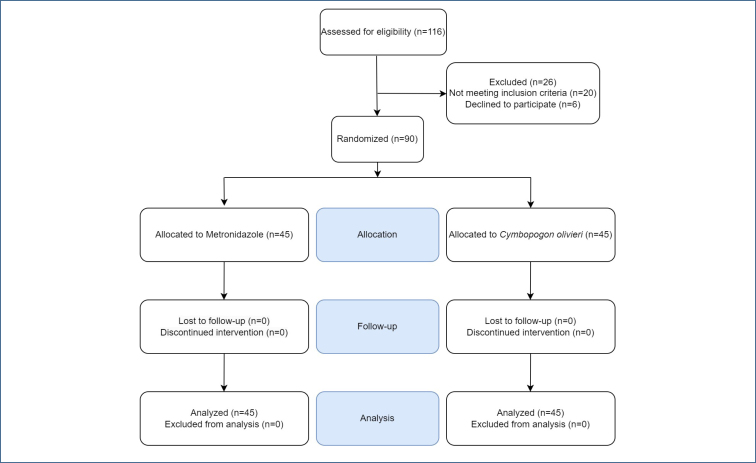
Flow diagram of the study.

A total of 116 patients were assessed for eligibility and 26 of them were excluded. At the end of the study, 90 women with BV, 45 of whom were in the *C. olivieri* group and 45 in the metronidazole group, completed the study. The study process is illustrated in Figure 1.

The findings indicate that there is no difference in any of the demographic factors between the metronidazole and *C. olivieri* groups. Patients who received *C. olivieri* had an average age and marriage average of 30.86 and 18.73 years, respectively, while patients who received metronidazole had an average age and marriage average of 32.60 and 18.93 years, respectively. Patients who received *C. olivieri* had 4.09, 3.35, and 0.73 pregnancies, children, and abortions, respectively. Patients who received metronidazole had 4.82, 4.3, and 0.57 pregnancies, children, and abortions, respectively. Table 2 shows the outcomes.

**Table 2:** Comparison of baseline demographic characteristics of the *Cymbopogon olivieri* and metronidazole groups before intervention.

Demographic characteristics	*Cymbopogon olivieri* Mean±SD	Metronidazole Mean±SD	p-value
Age	30.86±7.56	32.60±6.74	0.645
Age of marriage	18.73±3.21	18.93±2.81	0.438
Number of pregnancies	4.09±2.87	4.82±2.86	0.445
Number of children	3.35±2.52	4.3±2.68	0.138
Number of abortions	0.73±1.17	0.57±0.86	0.742

### Clinical outcomes

Clinical results are shown in Table 3. According to the McNemar test, the use of *C. olivieri* and metronidazole has significantly reduced the burning, itching, malodor, abnormal vaginal discharge, pH, clue cell, and positive whiff test (significance level less than 0.05). The results show that both treatments were effective in eliminating at least three out of four Amsel's criteria, and no statistically significant difference was found among them. The chi-square test shows that after taking the drugs *C. olivieri* and metronidazole, the number of improved patients in terms of burning, itching, malodor, abnormal vaginal discharge, pH, whiff test, and clue cell is not significantly different.

**Table 3:** Comparison of signs, symptoms, and Amsel's criteria before and after the test in *Cymbopogon olivieri* and metronidazole groups.

Variables		*Cymbopogon olivieri*			Metronidazole			p-values* (comparison of drugs after treatment)
		Before n (%)	After n (%)	p-value	Before n (%)	After n (%)	p-value	
Vaginal burning	No	0 (0)	42 (93.3)	<0.001	2 (4.4)	38 (84.4)	<0.001	0.180
	Yes	45 (100)	3 (6.7)		43 (95.6)	7 (15.6)		
Itching	No	0 (0)	43 (95.6)	<0.001	8 (17.8)	39 (86.7)	<0.001	0.138
	Yes	45 (100)	2 (4.4)		37 (82.2)	6 (13.3)		
Malodor	No	3 (6.7)	40 (88.9)	<0.001	9 (20)	41 (91.1)	<0.001	0.725
	Yes	42 (93.3)	5 (11.1)		36 (80)	4 (8.9)		
Abundant vaginal discharge	No	0 (0)	38 (84.4)	<0.001	0 (0)	36 (80)	<0.001	0.581
	Yes	45 (100)	7 (15.6)		45 (100)	9 (20)		
pH	<4.5	4 (8.9)	42 (93.3)	<0.001	0 (0)	40 (88.9)	<0.001	0.459
	>4.5	41 (91.1)	3 (6.7)		45 (100)	5 (11.1)		
Whiff test	Negative	3 (6.7)	40 (88.9)	<0.001	4 (8.9)	42 (93.3)	<0.001	0.459
	Positive	42 (93.3)	5 (11.1)		41 (91.1)	3 (6.7)		
Clue cell	Negative	5 (11.1)	40 (88.9)	<0.001	5 (11.1)	42 (93.3)	<0.001	0.459
	Positive	40 (88.9)	5 (11.1)		40 (88.9)	3 (6.7)		

* Chi-square test.

The results show that after taking *C. olivieri* product and metronidazole, variables of burning, itching, malodor, abnormal vaginal discharge, pH, whiff test, and clue cell are not significantly different from each other. Therefore, the effect of the herbal medicine of *C. olivieri* on the problems of patients with BV is the same as the effect of metronidazole.

## DISCUSSION

This study showed that *C. olivieri* is as effective as metronidazole in the treatment of BV. Based on the study of Azizian Sharme et al., *C. olivieri* methanolic extract had the highest amount of phenolic and flavonoid compounds and the essential oil analysis shows the main compounds were piperitone, 2-carene, and d-limonene. Phenolic, flavonoid, and terpenoid compounds have a high inhibitory effect against free radicals (antioxidant activity) and pathogenic pathogens (bacteria and fungi)^17^. The antibacterial impact of essential oils on Gram-positive bacteria is most likely due to the cytoplasm of the microbe releasing from the cell walls, which in effect causes the bacterium to become inactive. The basic mechanism of the effect of essential oils can be considered the inhibition of the synthesis of DNA, RNA, proteins, and polysaccharides^18^. Mahboubi M. et al.'s study showed that *C. olivieri* had an antibacterial effect on *Acinetobacter* sp., and even the antimicrobial effect of this plant was greater than that of other plants in that research^19^. The good effect of the plant on the symptoms and clinical criteria of Amsel seems to be due to its antibacterial activity. Some studies show that *Cymbopogon schoenanthu* has anti-inflammatory properties, which also confirm the improvement of the clinical symptoms of patients, including burning and itching^20^. Other similar studies have investigated the effect of other herbs or probiotics^21,22^ on BV. In the study of Baery et al., vaginal suppositories comprising the plants of *Tribulus terrestris, Myrtus commuis, Foeniculum vulgare*, and *Tamarindus indica* were used for treatment. The amount of abnormal vaginal discharge, Amsel criteria, pelvic pain, and cervical inflammation were significantly reduced in the group of herbal medicine and metronidazole (p=0.001). There was no statistically significant difference between the two groups of metronidazole and herbal medicine in any of the clinical symptoms or laboratory evaluations^23^. In the study of Alizadeh et al., the effect of *Hypericum perforatum L.* on BV was investigated and compared with metronidazole. In 10–12 days, the recovery rate was 82% in the *H. perforatum* group and 85% in the metronidazole group^24^. These studies show that plants can be used to treat BV along with antibiotics. Also, in this study, *C. olivieri* was good at treating BV. As a result, the treatment of BV by herbal medicine such as *C. olivieri* can alternatively be recommended. The limitations of this study included the limited time for follow-up, the low sample size, and the use of a single dose of the herbal formulation. It is suggested to conduct studies with larger sample sizes in this field and to follow up the patients after the study.

## CONCLUSION

This study demonstrates that *C. olivieri* is effective in lowering the clinical symptoms of BV. Its effect was comparable to that of metronidazole. These results are obtained due to its antimicrobial, anti-inflammatory, and antioxidant effects.
